# Effect of Adding Intermediate Layers on the Interface Bonding Performance of WC-Co Diamond-Coated Cemented Carbide Tool Materials

**DOI:** 10.3390/molecules28165958

**Published:** 2023-08-09

**Authors:** Junru Yang, Yanping Yue, Hao Lv, Baofei Ren, Yuekan Zhang

**Affiliations:** College of Mechanical and Electronic Engineering, Shandong University of Science and Technology, Qingdao 266590, China; yangjunru@sdust.edu.cn (J.Y.); yue171442@163.com (Y.Y.); lvhao1232023@163.com (H.L.); fei997283451@126.com (B.R.)

**Keywords:** diamond-coated cemented carbide, intermediate layer, first principle, interface adhesion work, charge distribution, density of states

## Abstract

The interface models of diamond-coated WC-Co cemented carbide (DCCC) were constructed without intermediate layers and with different interface terminals, such as intermediate layers of TiC, TiN, CrN, and SiC. The adhesion work of the interface model was calculated based on the first principle. The results show that the adhesion work of the interface was increased after adding four intermediate layers. Their effect on improving the interface adhesion performance of cemented carbide coated with diamond was ranked in descending order as follows: SiC > CrN > TiC > TiN. The charge density difference and the density of states were further analyzed. After adding the intermediate layer, the charge distribution at the interface junction was changed, and the electron cloud at the interface junction overlapped to form a more stable chemical bond. Additionally, after adding the intermediate layer, the density of states of the atoms at the interface increased in the energy overlapping area. The formant formed between the electronic orbitals enhances the bond strength. Thus, the interface bonding performance of DCCC was enhanced. Among them, the most obvious was the interatomic electron cloud overlapping at the diamond/SiC_C-Si_/WC-Co interface, its bond length was the shortest (1.62 Å), the energy region forming the resonance peak was the largest (−5–20 eV), and the bonding was the strongest. The interatomic bond length at the diamond/TiN_Ti_/WC-Co interface was the longest (4.11 Å), the energy region forming the resonance peak was the smallest (−5–16 eV), and the bonding was the weakest. Comprehensively considering four kinds of intermediate layers, the best intermediate layer for improving the interface bonding performance of DCCC was SiC, and the worst was TiN.

## 1. Introduction

Diamond-coated cemented carbide tools have the advantages of high strength, high hardness, low friction coefficient, etc. [1,2]. This makes them suitable for processing high-strength materials such as nonferrous metals and their alloys, special graphite, fiber, or ceramic-reinforced composites [3,4,5]. However, a graphite phase is formed at the interface due to the strong catalytic effect of the bonding phase of Co in the cemented carbide substrate [6], and thermal stresses between the diamond coating and the substrate are induced by the corresponding difference in the thermal expansion coefficient, deteriorating the interface bonding performance between the diamond coating and the substrate [7] and limiting the coated tool applicability.

Available methods used to mitigate this problem include cobalt-free treatment [8], changing the substrate composition [9], texturing the substrate surface [10,11,12], and adding intermediate layers [13,14,15,16]. Among them, adding an intermediate layers is currently a commonly used method to improve interface bonding performance. At present, commonly added intermediate layers include TiC, TiN, CrN, SiC, etc. Based on the acid etching of cobalt, Yang et al. [17] predeposited a TiN intermediate layer whose N content changed with gradients and prepared a diamond coating with good quality and adhesion. Liu et al. [18] reported that the TiC intermediate layer improved diamond films’ growth rate and adhesion force. Chandran et al. [19] added a CrN intermediate layer between the WC-6wt% Co cemented carbide substrate and the diamond coating, and an indentation test showed good adhesion of the CrN intermediate layer to the diamond coating and the cemented carbide substrate. Wang et al. [20] deposited a SiC intermediate layer on DCCC tools using the high current arc plasma chemical vapor deposition method, and the prepared DCCC tools had strong adhesion and excellent impact resistance.

Research on DCCC with intermediate layers has mainly focused on experiments, while its interface bonding mechanism at the atomic scale remains unclear. Given this, the current study constructs the interface model of diamond-coated WC-Co cemented carbide with the addition of TiC, TiN, CrN, and SiC intermediate layers. The model is based on the first principle, the Hohenberg–Kohn theorem, and the Kohn–Sham (KS) equation [21,22]. By calculating the interface adhesion work and analyzing the charge density difference and the density of states, the effects of different intermediate layers on the interface bonding properties of DCCC are explored. This study is of great significance in revealing the bonding mechanism of different intermediate layers on the interface of DCCC and optimizing the design of the interface structure of DCCC.

## 2. Results and Discussion

### 2.1. Interface Adhesion Work

The interface adhesion work is the reversible work per unit area required to separate the interface into two free surfaces. The greater the interface adhesion work, the more stable the interface structure and the better the interface bonding performance. The calculation formula of the interface adhesion work is as follows [23]:

(1) $$W_{ad}=\frac{E_{\alpha }+E_{\beta }-E_{\alpha /\beta }}{A_{\alpha /\beta }}$$

where *W_ad_* is the interface adhesion work, J/m^2^; *E_α_* and *E_β_* are the energies of the *α* and *β* surface configurations, respectively, eV; *E_α_*_/*β*_ is the constructed total energy of the *α*/*β* interface model, eV; and *A_α_*_/*β*_ is the interface area, Å^2^.

Through the single point energy calculation, the total energy *E_α_*_/*β*_ of each interface model after the geometry optimization and the energy of each surface model (*E_α_*) and (*E_β_*) were obtained. The adhesion work of each interface model was thus obtained by substituting the above data into Equation (1), as shown in Table 1.

From Table 1, the adhesion work of the diamond/graphite interface was minimal, namely, 0.028 J/m^2^. The adhesion work of the graphite/WC-Co interface was also low, namely, 2.758 J/m^2^. The adhesion work of the interface model after the addition of the intermediate layer was greater than that of the diamond/graphite/WC-Co interface model, which showed that the interface adhesion performance of DCCC was improved after the addition of TiC, TiN, CrN, and SiC intermediate layers.

The diamond coating and graphite layer in the figure are represented by DC and GL, respectively, in the following text.

**Figure 1:**
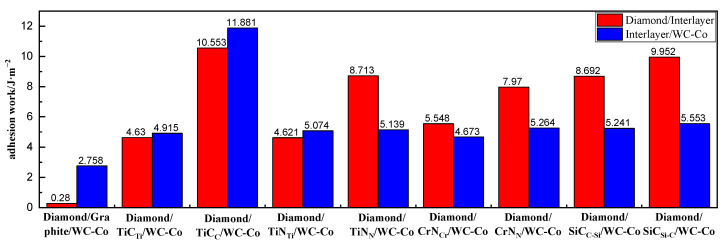
Adhesion work of the interface model.

Figure 1 shows the adhesion work of each interface model, comparing the adhesion work of different interface terminals of each intermediate layer, it was found that after adding the TiC intermediate layer, the most vulnerable interface model was DC/TiC_Ti_/WC-Co, and the interface with the lowest adhesion work was the DC/TiC_Ti_ interface, with a value of 4.63 J/m^2^_._ After adding the TiN intermediate layer, the most vulnerable interface model was DC/TiN_Ti_/WC-Co, and the interface with the lowest adhesion work was the DC/TiN_Ti_ interface, with a value of 4.621 J/m^2^. After adding the CrN intermediate layer, the most vulnerable interface model was DC/CrN_Cr_/WC-Co, and the interface with the lowest adhesion work was CrN_Cr_/WC-Co, with a value of 4.673 J/m^2^. After adding the SiC intermediate layer, the most vulnerable interface model was DC/SiC_C-Si_/WC-Co, and the interface with the lowest adhesion work was SiC_C-Si_/WC-Co, with a value of 5.241 J/m^2^.

To analyze the effect of different intermediate layers on the interface bonding performance of DCCC, interface models’ minimum interface adhesion work was used to characterize the overall interface bonding performance of the diamond coating and cemented carbide substrate.

The adhesion work values of the four most vulnerable interface models had the following descending order: DC/SiC_C-Si_/WC-Co > DC/CrN_Cr_/WC-Co >DC/TiC_Ti_/WC-Co > DC/TiN_Ti_/WC-Co. The respective improvement effects of four intermediate layers on the interface adhesion performance of DCCC ranked in descending order as SiC > CrN > TiC > TiN. Thus, the SiC and TiN layers had best and worst effects on the DCCC interface bonding strength, respectively.

### 2.2. Charge Density Difference Analysis

**Figure 2:**
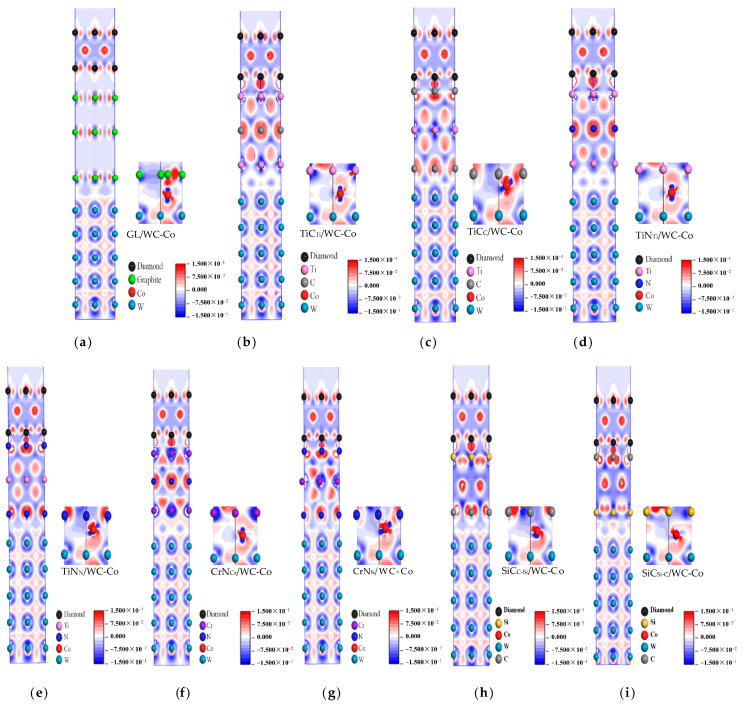
Charge density difference diagram of each interface model: (**a**) DC/GL/WC-Co; (**b**) DC/TiC_Ti_/WC-Co; (**c**) DC/TiC_C_/WC-Co; (**d**) DC/TiN_Ti_/WC-Co; (**e**) DC/TiN_N_/WC-Co; (**f**) DC/CrN_Cr_/WC-Co; (**g**) DC/CrN_N_/WC-Co; (**h**) DC/SiC_C-Si_/WC-Co; (**i**) DC/SiC_Si-C_/WC-Co.

The interface adhesion work analysis revealed that the intermediate layer improved the interface bonding performance between the diamond coating and cemented carbide substrate. To reveal the mechanism of the intermediate layer, the charge density distribution and the bonding between atoms at the interface were analyzed by the charge density difference diagram. The charge density difference diagram of each interface model is shown in Figure 2, where the red, blue, and white areas represent electron enrichment, electron loss, and a slight change in electron density, respectively.

Figure 2 and Table 2 show each interface model’s charge density difference diagram and the bond length at the interface, respectively. In Figure 2a, their analysis revealed no charge transfer between the C (graphite) atom and the C (DC) atom at the DC/GL interface, indicating poor diamond nucleation on the graphite substrate. In addition, there was no charge transfer between the layers of graphite, which was contributed by the van der Waals force. The Co atom and the graphite layer at the GL/WC-Co interface were also contributed by the van der Waals force, which was weak.

Figure 2b shows that there was a large amount of shared charge between Ti (TiC) atoms and C (DC) atoms at the DC/TiC_Ti_ interface, forming the covalent bond of Ti(TiC)-C(DC). An inevitable electron cloud overlap between Co atoms and Ti (TiC) atoms at the TiC_Ti_/WC-Co interface formed the Co-Ti (TiC) bond. In Figure 2c, there is a large amount of shared charge between the C (TiC) atom and the C (DC) atom at the DC/TiC_C_ interface, forming the C (TiC)-C (DC) covalent bond, whose bond length (1.45) was shorter than that of the Ti (TiC)-C (DC) bond (2.14) and whose bond strength was stronger than that of the Ti (TiC)-C (DC) bond. At the TiC_C_/WC-Co interface, there was an obvious electron cloud overlap between the Co atom and the C (TiC) atom, forming the Co-C (TiC) bond, whose bond length (1.97) was shorter than that of Co-Ti (TiC) (4.01) and whose bond strength was stronger than that of the Co-Ti (TiC) bond. Therefore, compared to the TiC_Ti_ terminal intermediate layer, the TiCc terminal intermediate layer had a better effect on improving the interface bonding performance.

Figure 2d shows that there was a large amount of shared charge between Ti (TiN) and C (DC) atoms at the DC/TiN_Ti_ interface, forming a Ti (TiN)-C (DC) covalent bond. At the TiN_Ti_/WC-Co interface, electron clouds overlapped between Co atoms and Ti (TiN) atoms, forming a Co-Ti (TiN) bond. In Figure 2e, there was a large amount of shared charge between N (TiN) atoms and C (DC) atoms at the DC/TiN_N_ interface, forming the N (TiN)-C (DC) covalent bond, whose bond length (1.59) was shorter than that of the Ti (TiN)-C (DC) bond (2.15) and whose bond strength was stronger than that of the Ti (TiN)-C (DC) bond. At the TiN_N_/WC-Co interface, there was an obvious electron cloud overlap between the Co atom and the N (TiN) atom, forming the Co-N (TiN) bond, whose bond length (3.97) was shorter than that of the Co-Ti (TiN) bond (4.11) and whose bond strength was stronger than that of the Co-Ti (TiN) bond. Therefore, compared to TiN_Ti_ terminal intermediate layer, the TiN_N_ terminal intermediate layer had a better effect on improving the interface bonding performance.

Figure 2f shows that there was a large amount of shared charge between Cr (CrN) atoms and C (DC) atoms at the DC/CrN_Cr_ interface, forming a Cr (CrN)-C (DC) covalent bond. At the CrN_Cr_/WC-Co interface, there was an inevitable overlap of electron clouds between Co atoms and Cr (CrN) atoms, forming a Co-Cr (CrN) bond. In Figure 2g, there was a large amount of shared charge between N (CrN) atoms and C (D) atoms at the DC/CrN_N_ interface, forming an N (CrN)-C (DC) covalent bond, whose bond length (1.69) was shorter than that of the Cr (CrN)-C (DC) bond (1.97) and whose bond strength was stronger than that of the Cr (CrN)-C (DC) bond. At the CrN_N_/WC-Co interface, there was an obvious electron cloud overlap between the Co atom and the N (CrN) atom, forming a Co-N (CrN) bond whose bond length (3.86) was shorter than that of the Co-Cr (CrN) bond (4.05) and whose bond strength was stronger than that of the Co-Cr (CrN) bond. Therefore, compared to the CrN_Cr_ terminal intermediate layer, the CrN_N_ terminal intermediate layer had a better effect on improving the interface bonding performance.

Figure 2h shows that there was a large amount of shared charge between Si (SiC) atoms and C (DC) atoms at the DC/SiC_C-Si_ interface, forming a Si (SiC)-C (DC) covalent bond. At the SiC_C-Si_/WC-Co interface, there was an inevitable overlap of electron clouds between Co atoms and C (SiC) atoms, forming a Co-C (SiC) bond. In Figure 2i, there was a large amount of shared charge between the C (SiC) atom and the C (DC) atom at the DC/SiC_Si-C_ interface, forming a C (SiC)-C (DC) covalent bond, whose bond length (1.51) was shorter than that of the Si (SiC)-C (DC) bond (1.62) and whose strength was stronger than that of the Si (SiC)-C (DC) ween the Co atom and the Si (SiC) atom, forming a Co-Si (SiC) bond, whose bond length (3.67) was shorter than that of the Co-C (SiC) bond (3.91) and whose bond strength was stronger than that of the Co-C (SiC) bond. Therefore, compared to the SiC_C-Si_ terminal intermediate layer, the SiC_Si-C_ terminal intermediate layer had a better effect on improving the interface bonding performance.

Comprehensively comparing the charge distribution, the bonding at the interface of the DC/TiC_C_/WC-Co interface model was the strongest after adding the intermediate layer. Among the four intermediate layers, the bonding between atoms at the interface of the DC/TiC_Ti_/WC-Co, DC/TiN_Ti_/WC-Co, DC/CrN_Cr_/WC-Co, and DC/SiC_C-Si_/WC-Co interface models were weak. Thus, the corresponding charge distribution was compared and analyzed. The results show that the overlap of atomic electron clouds at the interface of the diamond/SiC_C-Si_/WC-Co interface model was more pronounced. In addition, the bond length was the shortest, indicating that the bonding effect between atoms at the interface was the strongest. Therefore, the interface bonding performance of DCCC was the best after adding the SiC intermediate layer, while the bond length at the interface of the DC/TiN_Ti_/WC-Co interface model was the longest, indicating that the bonding effect between atoms at the interface was the weakest and the effect of improving the interface bonding performance was the worst.

### 2.3. Analysis of the Density of States

**Figure 3:**
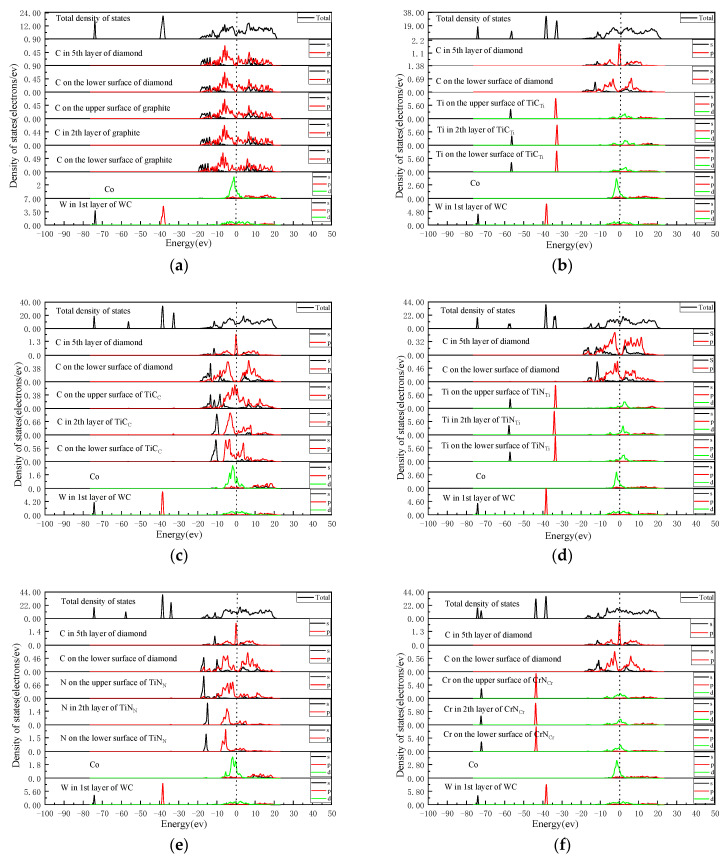

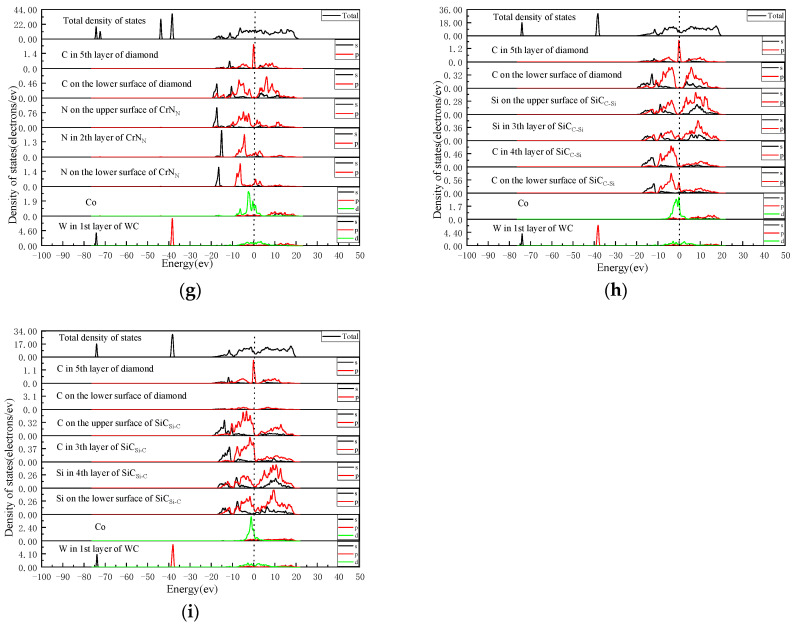
The total density of states and the partial density of states of each interface model: (**a**) DC/GL/WC-Co; (**b**) DC/TiC_Ti_/WC-Co; (**c**) DC/TiC_C_/WC-Co; (**d**) DC/TiN_Ti_/WC-Co; (**e**) DC/TiN_N_/WC-Co; (**f**) DC/CrN_Cr_/WC-Co; (**g**) DC/CrN_N_/WC-Co; (**h**) DC/SiC_C-Si_/WC-Co; (**i**) DC/SiC_Si-C_/WC-Co.

The density of states of each interface model was calculated to further explore the bonding nature between atoms at the interface. The total and partial density of the states of each interface model are shown in Figure 3.

As seen in Figure 3a, there was no resonance peak between C (DC) atoms and C (GL) atoms at the DC/GL interface, indicating that there was no bonding between C (GL) atoms and C (DC) atoms. The density of states of Co atoms and C (GL) atoms at the GL/WC-Co interface had a resonance in the energy range of −5–20 eV, which was mainly contributed by the valence electrons of Co-d and C-p. However, the density of states of C (GL) atoms in the overlapping region was low, indicating the low interaction between Co atoms and C (GL) atoms.

In Figure 3b, the density of states of Ti (TiC) and C (DC) atoms at the DC/TiC_Ti_ interface formed a resonance peak in the energy region of −5–20 eV, which was mainly contributed by the Ti-d orbital and C-s orbital, leading to bonding between Ti (TiC) atoms and C (DC) atoms. At the TiC_Ti_/WC-Co interface, the density of states of Co atoms and Ti (TiC) atoms formed a resonance peak in the energy range of −5–20 eV, which was mainly contributed by the Co-d orbital and Ti-d orbital, leading to bonding between the Co atom and the Ti (TiC) atom. The density of states of Ti (TiC) atoms in the overlapping region was greater than that of C (GL) atoms. The bonding force between Co and Ti (TiC) atoms was stronger than between Co and C (GL) atoms. In Figure 3c, the density of states of C (TiC) atoms and C (DC) atoms at the DC/TiC_C_ interface formed a resonance peak in the energy region of −20–20 eV, which was contributed by the C (TiC)-p orbital and the C (DC)-p orbital, leading to the bonding between C (TiC) atoms and C (DC) atoms. The larger the energy region of orbital resonance is, the stronger the bonding. The energy region of the C (TiC)-C (DC) bond was greater than that of the Ti (TiC)-C (DC) bond. Therefore, the C (TiC)-C (DC) bond was stronger than the Ti (TiC)-C (DC) bond. The density of states of Co atoms and C (TiC) atoms at the TiC_C_/WC-Co interface formed resonance peaks in the energy region of −7–20 eV, which were contributed by Co-d, Co-p, and C-p orbitals, respectively. The bonding of Co atoms with C (TiC) atoms was stronger than that of Co-Ti (TiC) atoms. Therefore, for the TiC_Ti_ terminal intermediate layer, the TiCc terminal intermediate layer had a better effect on improving the interface bonding performance.

As seen in In Figure 3d, the density of states of Ti (TiN) and C (DC) atoms at the DC/TiN_Ti_ interface formed a resonance peak in the energy region of −5–16 eV, which was mainly contributed by the Ti-d orbital and the C-p orbital, leading to bonding between Ti (TiN) atoms and C (DC) atoms. At the TiN_Ti_/WC-Co interface, the density of states of Co atoms and Ti (TiN) atoms formed a resonance peak in the energy region of −5–5 eV, which was mainly contributed by the Co-d orbital and the Ti-d orbital, leading to bonding between Co and Ti (TiN) atoms. The density of states of Ti (TiN) atoms in the overlapping region exceeded that of the C (GL) atoms. The force bond between Co and Ti (TiN) atoms was stronger than that between Co and C (GL) atoms. As seen in Figure 3e, the density of states of N (TiN) atoms and C (DC) atoms at the DC/TiN_N_ interface formed a resonance peak in the energy region of −15–15 eV, which was contributed by N-s, N-p orbitals, and C-s, C-p orbitals, leading to the bonding between N (TiN) and C (DC) atoms. The corresponding orbital resonance energy area was large. The corresponding bond strength was stronger than that of the Ti (TiN)-C (DC) bond. The density of states of Co atoms and N (TiN) atoms at the TiN_N_/WC-Co interface formed a resonance peak in the energy region of −7–20 eV, mainly due to the orbital hybridization of Co-d and N-p, leading to bonding between the Co atoms and N (TiN) atoms. The corresponding orbital resonance energy region was large. The corresponding bond strength was stronger than that of the Co-Ti (TiN) bond. Therefore, for the TiN_Ti_ terminal intermediate layer, the TiN_N_ terminal intermediate layer had a better effect on improving the interface bonding performance.

As seen in Figure 3f, the density of states of Cr (CrN) and C (DC) atoms at the DC/CrN_Cr_ interface formed a resonance peak in the energy region of −16–10 eV, which was mainly contributed by Cr-d, Cr-p, and C-p orbitals, leading to bonding between Cr (CrN) atoms and C (DC) atoms. The density of states of Co atoms and Cr (CrN) atoms at the CrN_Cr_/WC-Co interface formed a resonance peak in the energy region of −5–5 eV, which was mainly contributed by the Co-d orbital and the Cr-d orbital, leading to bonding between the Co atoms and the Cr (CrN) atoms. The density of states of Cr (CrN) atoms in the overlapping region was greater than that of C (GL) atoms. The bonding force between Co and Cr (CrN) atoms was stronger than between Co and C (GL) atoms. In Figure 3g, the density of states of N (CrN) atoms and C (DC) atoms at the DC/CrN_N_ interface formed a resonance peak in the energy region of −20–15 eV, which was contributed by N-s, N-p orbitals, and C-s, C-p orbitals, leading to the bonding between N (CrN) atoms and C (DC) atoms. The corresponding orbital resonance energy area was large. The corresponding bond strength was stronger than that of the Cr (CrN)-C (DC) bond. The density of states of Co atoms and N (CrN) atoms at the CrN_N_/WC-Co interface formed a resonance peak in the energy region of −10–5 eV, which was mainly contributed by the Co-d and N-p orbitals, leading to bonding between Co and N (CrN) atoms. The corresponding orbital resonance area was large. The corresponding bond strength was stronger than that of the Co-Cr (CrN) bond. Therefore, for the CrN_Cr_ terminal intermediate layer, the CrN_N_ terminal intermediate layer had a better effect on improving the interface bonding performance.

As seen in Figure 3h, the density of states of Si (SiC) atoms and C (DC) atoms at the DC/SiC_C-Si_ interface formed a resonance peak in the energy region of −20–16 eV, which was mainly contributed by Si-s, Si-p orbitals, and C-s, C-p orbitals, leading to bonding between Si (SiC) atoms and C (DC) atoms. The density of states of Co and C (SiC) atoms at the SiC_C-Si_/WC-Co interface formed a resonance peak in the energy region of −5–20 eV, which was mainly contributed by the Cod orbital and the C-p orbital, leading to bonding between Co atoms and C (SiC) atoms. The density of states of C (SiC) atoms in the overlapping region was greater than that of C (GL) atoms. The bonding force between Co atoms and C (SiC) atoms was stronger than that between Co atoms and C (GL) atoms. In Figure 3i, the density of states of C (SiC) atoms and C (DC) atoms at the DC/SiC_Si-C_ interface formed a resonance peak in the energy region of −20–18 eV, which was mainly contributed by the C-s orbital and the C-p orbital, leading to bonding between C (SiC) atoms and C (DC) atoms. The corresponding orbital resonance energy region was large. The corresponding bond strength was stronger than that of the Si (SiC)-C (DC) bond. The density of states of Co and Si (SiC) atoms at the SiC_Si-C_/WC-Co interface formed a resonance peak in the energy range of −5–20 eV, mainly contributed by the Co-d orbital and the Si-p orbital, leading to bonding between Co and Si (SiC) atoms. The corresponding density of states was high. The corresponding bond strength was stronger than that of the Co-C (SiC) bond. Therefore, for the SiC_C-Si_ terminal intermediate layer, the SiC_Si-C_ terminal intermediate layer had a better effect on improving the interface bonding performance.

The energy region of the density of states orbital resonance in the DC/TiN_N_/WC-Co interface model was the largest, and the bonding at the interface was the strongest. Among the four kinds of intermediate layers, the DC/TiC_Ti_/WC-Co, DC/TiN_Ti_/WC-Co, DC/CrN_Cr_/WC-Co, and DC/SiC_C-Si_/WC-Co interface models had a minor energy region that formed resonance peaks and weak stability of the interface structure. Therefore, the densities of states of these four interface models were compared and analyzed. The results show that the energy region of the density of states of atoms that formed the resonance peak at the interface of the DC/SiC_C-Si_/WC-Co interface model was the largest. Meanwhile, the intensity of interatomic bonding was the strongest. Therefore, the interface bonding performance of DCCC was the best after adding the SiC intermediate layer, while the energy region of orbital resonance in the DC/TiN_Ti_/WC-Co interface model was the smallest, indicating the weak interatomic bonding effect at the interface and the worst effect of improving the interface bonding performance.

The comprehensive analysis showed that the interface bonding property of DCCC significantly improved after adding intermediate layers. To verify the improvement effect of different intermediate layers on the interface bonding properties of DCCC, the most unstable interface models of each intermediate layer were compared. Through the comprehensive comparison of the interface adhesion work, the charge density difference, and the density of states, the improvement effects of four intermediate layers on the interface bonding properties of DCCC were ranked in descending order as follows: SiC > CrN > TiC > TiN. Thus, the SiC and TiN layers had the best and worst effects on the DCCC interface bonding strength, respectively.

These findings were further compared with experimental results of previous studies. Thus, Liu et al. [24] prepared an intermediate layer between a cemented carbide substrate and a diamond coating by the hot-filament chemical vapor deposition method. The binding force between the diamond film and WC-6wt% Co cemented the membrane separation area quantitatively evaluated carbide substrate under the static loading of 9.8 N in the indentation experiment. The membrane separation area without an intermediate layer was 448 μm^2^, and the areas with TiN, TiC, and CrN intermediate layers were 394, 328, and 290 μm^2^, respectively. The results show that among the three intermediate layers, the improvement effect on the interface bonding performance of DCCC had the following descending order: CrN > TiC > TiN.

In addition, Chandran et al. [19] used the indentation method to test the interface bonding performance of a DCCC tool with the CrN intermediate layer added to the YG6 substrate. When the coating cracked, the indentation pit unit area bore a load of 198 N. Wang et al. [20] used the indentation method to test the interface bonding performance of a DCCC tool with a SiC intermediate layer added to the YG6 substrate. When the coating cracked, the indentation pit unit area bore a load of 477 N. The improvement effect of adding the SiC intermediate layer on the interface bonding performance of the DCCC was better than that of CrN intermediate layer.

Overall, the simulation analysis results in this work are consistent with the available experimental research results in the references.

## 3. Materials and Methods

### 3.1. Material Interface Model Construction Process

Yefei Li et al. [25] reported that the W atom terminal’s WC (001) surface was the most stable. Through experimental research, Jing Liu [26] proved that SiC films grew preferentially in the (111) plane on cemented carbide. Hongwu Liu [27] proved that diamond films grew mainly in the <111> direction. According to crystal growth theory, graphite grows preferentially along the base plane (001) to form a lamellar structure. The lower the energy, the more stable the structure. Therefore, when different crystals combine to form an interface, the surface with low surface energy is usually selected as the interface. The TiC, TiN, and CrN surfaces have a face-centered cubic structure, and the surface with the lowest surface energy is densely arranged (111). Therefore, this study incorporated WC (001), GL (001), SiC (111), TiC (111), TiN (111), CrN (111), and Diam (111) crystal planes into the constructed interface model.

In addition, the more atomic layers the model has, the more similar it is to the volume phase characteristics. Therefore, in the process of building the model, while considering computer computing performance, in order to maintain the volume phase characteristics of the surface model, it is necessary to determine the number of atomic layers for each model. The method is to compare the surface energy of different atomic layers of the model to determine the appropriate number of atomic layers for the model. The formula for calculating surface energy is as follows:

(2) $$E_{surf}=\frac{E_{slab}-(\frac{N_{slab}}{N_{bulk}})E_{bulk}}{2A}$$

where, $E_{surf}$ is the surface energy on the crystal plane, J/m^2^;$\;E_{slab}$ is the surface energy, eV; $E_{bulk}$ is the energy of the crystal cell, eV; $N_{slab}/N_{bulk}$ is the ratio of the number of surface atoms to the number of crystal cell atoms; and A is the crystal surface area, Å^2^.

We calculated the surface energy of surface models with different atomic layers, as shown in Table 3 and Table 4.

From Table 3 and Table 4, it can be seen thatwhen the WC(001)_W_ crystal plane has nine atomic layers, the SiC(111) and Diamond(111) crystal planes have six layers, and the TiC(111)_Ti_, TiC(111)_C_, TiN(111)_Ti_, TiN(111)_N_, CrN(111)_Cr_, and CrN(111)_N_ crystal planes have seven atomic layers, and the surface energy of each crystal surface model begins to converge. Graphite (001) has extremely low surface energy and an extremely stable graphite structure. Therefore, we selected nine-layer WC(001)_W_, six-layer SiC(111), six-layer Diamond(111), three-layer Graphite (001), and seven-layer TiC(111)_Ti_, TiC(111)_C_, TiN(111)_Ti_, TiN(111)_N_, CrN(111)_Cr_, and CrN(111)_N_ surface models, and used the Build layers function in Material Studio 2020 software to construct relevant models. When the diamond coating was deposited on the WC-Co cemented carbide’s gold substrate, the binding phase of Co in the cemented carbide quickly diffused to the interface between the coating and the substrate under high temperature [28]. Mikael et al. [29] found that Co could simulate the surface structure of cemented carbide by replacing the C atom on the WC (001) surface. Because the diamond coating had good adhesion on the WC-Co substrate when the content of Co in the cemented carbide was 6 wt% [30], WC-6wt% Co cemented carbide was selected as the substrate. The content of Co was calculated as follows:

(3) $$w_{\mathrm{Co}}=\frac{n_{\mathrm{Co}}A_{\mathrm{rCo}}}{n_{\mathrm{Co}}A_{\mathrm{rCo}}+n_{W}A_{\mathrm{rW}}+n_{C}A_{\mathrm{rC}}}\times 100\%$$

where *w*_Co_ is the Co content, wt%; *n*_Co_ is the number of Co atoms; *A*_rCo_ is the relative atomic mass of Co atoms, 58.93; *n*_W_ is the number of W atoms; *A*_rW_ is the relative atomic mass of W atoms, 183.84; *n*_C_ is the number of C atoms; and *A*_rC_ is the relative atomic mass of C atoms, 12.01. The content of Co was approximately assessed as 6 wt% via Equation (3).

**Figure 4:**
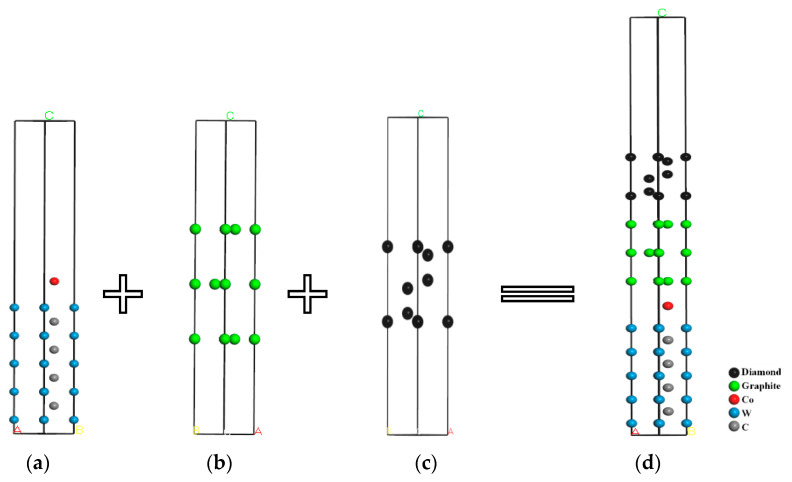
The construction process of the DC/GL/WC-Co interface model: (**a**) WC-Co; (**b**) GL; (**c**) DC; (**d**) DC/GL/WC-Co.

When the diamond coating was directly deposited on the cemented carbide, a graphite phase layer was formed between the coating and the substrate. Thus, the diamond/graphite/WC-Co interface model was constructed to represent DCCC. The construction process is shown in Figure 4. As shown in Figure 4a, the surface of the cemented carbide was characterized by the replacement of the C atoms on the WC (001) surface by the Co atoms at the interface between the coating and the substrate. A, B, and C are used to show the direction of vacuum layer, as shown in Figure 4.

According to the same construction process, considering that the surfaces of TiC (111), TiN (111), CrN (111), and SiC (111) had two types of terminal atoms, the interface models of DC/TiC_C_/WC-Co, DC/TiC_Ti_/WC-Co, DC/TiN_N_/WC-Co, DC/TiN_Ti_/WC-Co, DC/CrN_N_/WC-Co, DC/CrN_Cr_/WC-Co, DC/SiC_C-Si_/WC-Co, and DC/SiC_Si-C_/WC-Co were constructed. In addition, the interface mismatch degree during the construction process of the above model is less than 8%, and the constructed models have a certain degree of stability.

### 3.2. Material Interface Model Optimization

**Figure 5:**
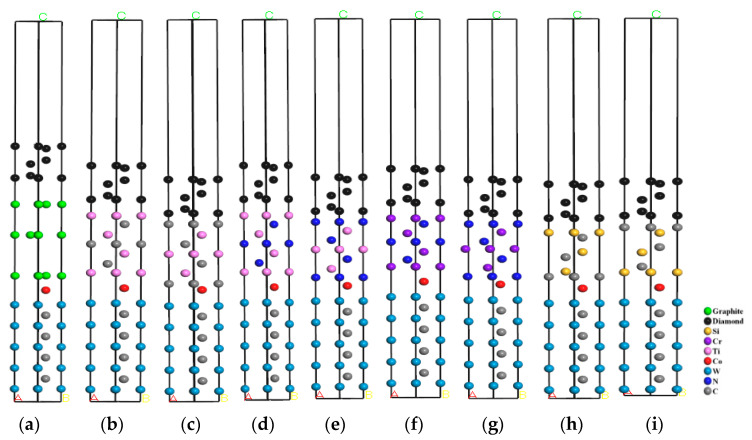
The interface models after geometry optimization: (**a**) DC/GL/WC-Co; (**b**) DC/TiC_Ti_/WC-Co; (**c**) DC/TiC_C_/WC-Co; (**d**) DC/TiN_Ti_/WC-Co; (**e**) DC/TiN_N_/WC-Co; (**f**) DC/CrN_Cr_/WC-Co; (**g**) DC/CrN_N_/WC-Co; (**h**) DC/SiC_C-Si_/WC-Co; (**i**) DC/SiC_Si-C_/WC-Co.

When analyzing based on first principles, the Castep module of Material Studio 2020 software was used for simulation calculations. The GGA-PBE exchange correlation functional was used. The cutoff energy was selected as 400 eV. The interaction between valence electrons and ionic realms was described by the ultrasoft pseudopotential. The K-point with the “fine precision” setting was selected. The BFGS algorithm was adopted to obtain a stable lattice configuration. The self-consistent iteration method was used for the total energy calculation. The self-consistent convergence conditions were as follows: the total energy convergence standard of the system was 1.0 × 10^−5^ eV/atom, the convergence standard of the interatomic interaction force was 0.03 eV/Å, the convergence standard of internal crystal stress was 0.05 GPa, and the convergence standard of maximum atomic displacement was 1.0 × 10^−3^ Å. Under the above parameter settings, geometry optimization was carried out for each interface model. The interface models after the geometry optimization are shown in Figure 5. A, B, and C in the figure represent the direction of the model.

## 4. Conclusions

Based on the first principle, the effects of TiC, TiN, CrN, and SiC intermediate layers on the interface bonding properties of diamond-coated WC-Co cemented carbide (DCCC) were studied. Interface models of DC/GL/WC-Co, DC/TiC_Ti_/WC-Co, DC/TiC_C_/WC-Co, DC/TiN_Ti_/WC-Co, DC/TiN_N_/WC-Co, DC/CrN_Cr_/WC-Co, DC/CrN_N_/WC-Co, DC/SiC_C-Si_/WC-Co, and DC/SiC_Si_-_C_/WC-Co of WC-6wt%Co cemented carbide tool materials without intermediate layers and with different interface terminals, such as intermediate layers of TiC, TiN, CrN, and SiC, were constructed. The adhesion work of the interface models was calculated. The electronic structure and the density of states was analyzed, and the main conclusions were as follows:

(1) The adhesion work analysis of the interface models showed that compared with the interface model with no intermediate layers, the interface adhesion work between the diamond coating (DC) and WC-Co cemented carbide substrate increased significantly after the addition of TiC, TiN, CrN, and SiC intermediate layers; namely, the interface bonding performance of DCCC increased after the addition of the intermediate layer. Among the interface models with the TiC intermediate layer, DC/TiC_Ti_/WC-Co had the lowest adhesion work, with a value of 4.630 J/m^2^. Among the interface models with the TiN intermediate layer, DC/TiN_Ti_/WC-Co had the lowest adhesion work, with a value of 4.621 J/m^2^. Among the interface models with the CrN intermediate layer, DC/CrN_Cr_/WC-Co had the lowest adhesion work, with a value of 4.673 J/m^2^. Among the interface models with the SiC intermediate layer, DC/SiC_C-Si_/WC-Co had the lowest adhesion work, with a value of 5.241 J/m^2^. The adhesion work values of the above four interface models were ranked in descending order as DC/SiC_C-Si_/WC-Co > DC/CrN_Cr_/WC-Co > DC/TiC_Ti_/WC-Co > DC/TiN_Ti_/WC-Co. The improvement effects of four intermediate layers on the interface bonding properties of DCCC were ranked as SiC > CrN > TiC > TiN.
(2) The charge density difference analysis showed that DCCC without intermediate layers had no charge transfer and no bonding between the atoms at the DC/GL interface and at the GL/WC-Co interface. Van der Waals forces combined the atoms at the interface with poor interface bonding performance. After adding the intermediate layers, the electron cloud between atoms at the interface overlapped to form a more stable chemical bond. Thus, the interface bonding performance was improved. The charge distributions of four interface models with a weak bonding effect after adding different intermediate layers were compared and analyzed. It was found that the charge overlap of atoms at the interface of the diamond/SiC_C-Si_/WC-Co interface model was significant, with the shortest bond length of 1.62 Å. The corresponding interatomic bonding effect at the interface was strong, and the interface bonding performance was the best. The corresponding bond length at the interface of the DC/TiN_Ti_/WC-Co interface model was the longest, namely 4.11 Å. Thus, the corresponding interatomic bonding effect at the interface was weak, indicating the worst effect on improving the interface bonding performance.
(3) The analysis of the density of states revealed that the density of states at the interface in DCCC without intermediate layers was low, and there were no formed resonance peaks. The interaction between atoms was weak. After adding the intermediate layer, resonance peaks were formed between atoms at the interface. The density of states of the atoms in the energy overlap region increased, enhancing the bonding force between the atoms at the interface and improved the interface bonding performance. After comparing and analyzing the density of states of four interface models with weak interfacial atomic forces after adding intermediate layers, it was shown that the energy region of the resonance peak formed by the density of states of the atoms at the interface of the DC/SiC_C-Si_/WC-Co interface model was the largest (−5–20 eV). The interatomic bonding strength was the strongest, and the interface bonding performance was the best. The energy region of the orbital resonance of the DC/TiN_Ti_/WC-Co interface model was the smallest (−5–16 eV). The bonding between the atoms at the interface was the weakest, with the worst effect on improving the interface bonding performance.

## Figures and Tables

**Table 1:** The energy and the adhesion work of each interface model.

Interface Models	Interface	*E_α_*/eV	*E_β_*/(eV)	*E_α/β_*/(eV)	*A_α/β_*/(Å^2^)	*W_ad_*/(J/m^2^)
Diamond/Graphite/WC-Co	Diamond/Graphite	−920.966	−12,254.485	−13,175.464	7.38	0.028
	Graphite/WC-Co	−1842.669	−11,331.522	−13,175.464		2.758
Diamond/TiC_Ti_/WC-Co	Diamond/TiC_Ti_	−920.733	−18,214.304	−19,137.174		4.630
	TiC_Ti_/WC-Co	−7803.415	−11,331.491	−19,137.174		4.915
Diamond/TiC_C_/WC-Co	Diamond/TiC_C_	−920.509	−16,762.721	−17,688.098		10.553
	TiC_C_/WC-Co	−6351.349	−11,331.268	−17,688.098		11.881
Diamond/TiN_Ti_/WC-Co	Diamond/TiN_Ti_	−920.719	−18,568.229	−19,491.080		4.621
	TiN_Ti_/WC-Co	−8157.288	−11,331.451	−19,491.080		5.074
Diamond/TiN_N_/WC-Co	Diamond/TiN_N_	−920.586	−17,234.426	−18,159.032		8.713
	TiN_N_/WC-Co	−6825.187	−11,331.474	−18,159.032		5.139
Diamond/CrN_Cr_/WC-Co	Diamond/CrN_Cr_	−920.696	−22,017.431	−22,940.687		5.548
	CrN_Cr_/WC-Co	−11,607.038	−11,331.493	−22,940.687		4.673
Diamond/CrN_N_/WC-Co	Diamond/CrN_N_	−920.601	−19,820.510	−20,744.788		7.970
	CrN_N_/WC-Co	−9410.965	−11,331.394	−20,744.788		5.264
Diamond/SiC_C-Si_/WC-Co	Diamond/SiC_C-Si_	−920.699	−12,117.603	−13,042.312		8.692
	SiC_C-Si_/WC-Co	−1708.362	−11,331.532	−13,042.312		5.241
Diamond/SiC_Si-C_/WC-Co	Diamond/SiC_Si-C_	−920.621	−12,117.240	−13,042.452		9.952
	SiC_Si-C_/WC-Co	−1708.763	−11,331.532	−13,042.452		5.553

**Table 2:** Bond length at the interface.

Interlayer	Graphite	TiCTi	TiCC	TiNTi	TiNN	CrNCr	CrNN	SiCC-Si	SiCSi-C
Bond length of interface at diamond/interlayer (Å)	4.05	2.14	1.45	2.15	1.59	1.97	1.69	1.62	1.51
Bond length of interface at interlayer/WC-Co (Å)	5.06	4.01	1.97	4.11	3.97	4.05	3.86	3.91	3.67

**Table 3:** Surface energies of surface models with different atomic layers.

Atomic Layer Number	WC(001)_W_ Surface Energy/J·m^−2^	TiC(111)_Ti_ Surface Energy/J·m^−2^	TiC(111)_C_ Surface Energy/J·m^−2^	TiN(111)_Ti_ Surface Energy/ J·m^−2^	TiN(111)_N_ Surface Energy/ J·m^−2^	CrN(111)_Cr_ Surface Energy/ J·m^−2^	CrN(111)_N_ Surface Energy/ J·m^−2^
3	3.356	1.798	7.532	1.966	4.513	3.287	3.149
5	3.460	1.826	7.791	2.108	4.017	3.458	2.969
7	3.431	1.892	8.069	2.034	3.910	3.427	2.915
9	3.414	1.935	8.060	2.029	3.922	3.423	2.912
11	3.409	1.952	8.040	2.002	3.957	3.422	2.905

**Table 4:** Surface energies of surface models with different atomic layers.

Atomic Layer Number	Diamond (111) Surface Energy/ J·m^−2^	SiC(111) Surface Energy/ J·m^−2^	Atomic Layer Number	Graphite (001) Surface Energy/J·m^−2^
4	3.356	1.798	1	0.0004
6	3.460	1.826	2	0.0012
8	3.431	1.892	3	0.0026
10	3.414	1.935	4	0.0037
12	3.409	1.952	5	0.0057

## Data Availability

Data sharing not applicable.

## Sample Availability

No samples were involved in this study.
